# The Efficacy of Surgical Resection versus Radiofrequency Ablation for the Treatment of Single Hepatocellular Carcinoma: A SEER-Based Study

**DOI:** 10.1155/2023/1269504

**Published:** 2023-02-21

**Authors:** Fang Wu, Chao Wei, Shicun Zhang, Shanshan Jia, Jidong Zhang

**Affiliations:** ^1^Department of Gastroenterology, School of Clinical Medical, Jiamusi University, Jiamusi, 154007, Heilongjiang Province, China; ^2^Department of Medical Oncology, The Seventh Hospital of Qiqihar, Qiqihar, 161000, Heilongjiang Province, China; ^3^Department of Neurology, The Second Affiliated Hospital of Qiqihar Medical University, Qiqihar, 161000, Heilongjiang Province, China; ^4^Department of Gastroenterology, The First Hospital of Qiqihar, Qiqihar, 161000, Heilongjiang Province, China

## Abstract

**Background:**

There is controversy regarding whether patients with single hepatocellular carcinoma (HCC) should be offered radiofrequency ablation (RFA) as a first-line treatment option. Thus, this study compared overall survival after surgical resection (SR) and RFA for single HCC.

**Methods:**

The Surveillance, Epidemiology, and End Results (SEER) database was used for this retrospective study. The study included 30- to 84-year-old patients diagnosed with HCC from 2000 to 2018. Selection bias was reduced via propensity score matching (PSM). The study compared the overall survival (OS) and cancer-specific survival (CSS) of patients with single HCC who were treated with SR and RFA.

**Results:**

Before and after PSM, the median OS and median CSS were significantly longer in the SR group than in the RFA group (*p* < 0.05). In the subgroup analysis, the median OS and median CSS for male and female patients with male and female patients with tumor sizes <3, 3–5, and>5 cm, age at diagnosis between 60 and 84 years, and grades I–IV tumors were longer than in the SR group than in the RFA group (*p* < 0.05). Similar results were reported for patients who received chemotherapy (*p* < 0.05). Univariate and multivariate analyses revealed that compared with RFA, SR was an independent favorable factor for OS and CSS (*p* < 0.05) before and after PSM.

**Conclusion:**

Patients with SR who had a single HCC showed higher OS and CSS compared with patients who received RFA. Hence, SR should be used as a first-line treatment in cases of single HCC.

## 1. Introduction

According to the 2018 Global Cancer Statistics, hepatocellular carcinoma (HCC) was the sixth most prevalent cancer in the world and the fourth leading cause of cancer-related deaths [1]. Worldwide, the number of HCC deaths is approximately the same as the incidence, with the incidence being higher among men. A liver cancer diagnosis occurs in 1 in 45 men and in 1 in 113 women before age 79 years [2]. A history of chronic alcohol abuse, non-alcoholic fatty liver disease, cirrhosis, or hepatitis B or C virus infection is most commonly associated with HCC development [3], and HCC has a poor prognosis historically. The current Barcelona-Clinic Liver Cancer staging system recommends liver transplantation (LT), surgical resection (SR), and radiofrequency ablation (RFA) as treatment methods for HCC patients with single tumors <5 cm or with three or less tumors <3 cm, without lymph node invasion or distant metastasis [4]. For early single HCC, LT is the best treatment. However, since donors are in short supply, both SR and RFA are considered effective treatments [5].

Regarding treatment for very early hepatocellular carcinomas, it is unclear which treatment is more effective between SR and RFA. Previous studies have shown that RFA is as effective as SR for treating small liver tumors [6–8]. The results of recent studies indicate that SR is associated with a better overall survival rate (OS) than RFA for patients with a single small HCC [9, 10]. However, it is unclear which therapy provides a better prognosis for patients with single HCC. Our study aimed to compare overall survival after SR or RFA for single HCC.

## 2. Materials and Methods

### 2.1. Patients

**Figure 1:**
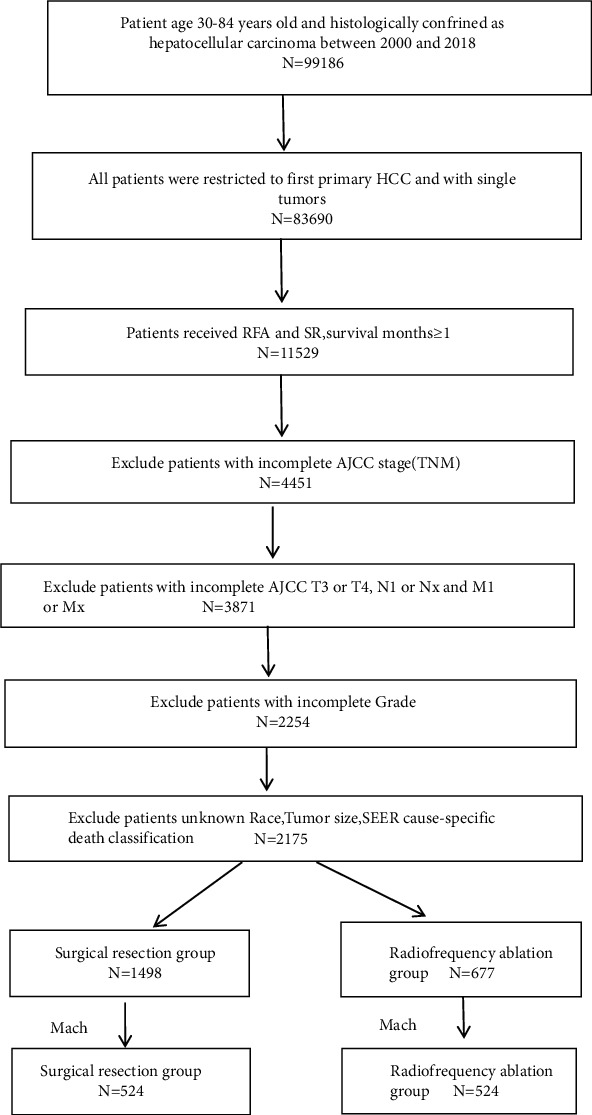
Flowchart of patient selection. HCC: hepatocellular carcinoma; RFA: radiofrequency ablation; SR: surgical resection. AJCC: American Joint Committee on Cancer (7th); SEER: the surveillance, epidemiology, and end results.

Data from the Surveillance, Epidemiology, and End Results (SEER)∗stat software (version 8.4.0) were used for the study after SEER-approval (the reference ID: 23587-Nov2020). Patients with the relevant site code (C22.0) and pathological diagnosis (ICD-O-3 histology codes: 8170-8175) were included in the analysis. All cases were restricted to the first primary HCC. The surgery codes used were: RFA-16 and SR-20 to 25, 30, 36, 37, 50, 51, and 52. The inclusion criteria were as follows: (1) diagnosis of the patient was between 2000 and 2018; (2) patient age 30–84 years; (3) presence of a single tumor that had not invaded the lymph nodes or spread to distant sites; (4) availability of complete survival data; and (5) history of RFA or SR. The exclusion criteria comprised (1) incomplete AJCC 7th TNM stage; (2) AJCC stages T3 or T4, N1 or NX, and M1 or MX; (3) incomplete grade; (4) unknown patient race; (5) unknown tumor size; and (6) missing/unknown cause-specific death classification (Figure 1).

### 2.2. Statistical Analyses

We extracted patient information from the SEER database. We converted all continuous variables to categorical variables were analyzed using the chi-square test or Fisher exact test. The median OS and median cause-specific survival (CSS) were determined using Kaplan–Meier survival curves and compared with the log-rank test. Before and after propensity score matching (PSM), Cox-proportional hazards model was used to identify OS and CSS predictors. Multivariate analysis was conducted using variables with *p*-values <0.1 in the univariate analysis. Age at diagnosis, sex, race, tumor size, grade, SEER stage, chemotherapy, and AJCC T stage were included into the PSM analysis. We set the optimal caliper at 0.01, and a total of 524 pairs of matched nearest neighbors were found. Values of *p* < 0.05 were considered to indicate statistical significance in all tests, which were two-sided. Statistics were analyzed using SPSS v22.0 (IBM, Armonk, NY, USA).

## 3. Results

### 3.1. Baseline Characteristics

The study included 2175 (1587 male and 588 female) patients, with 677 in the RFA group and 1498 in the SR group. Prior to PSM, the RFA group had more white patients and tumors sized <3 cm compared to the SR group (*p* < 0.001). The SR group had more cases without chemotherapy, and more moderately differentiated and more localized cancer cases than the RFA group (*p* < 0.001) (Table 1). After PSM, the characteristics of the two groups did not significantly differ (all *p* > 0.05) (Table 1).

### 3.2. Efficacy

**Figure 2:**
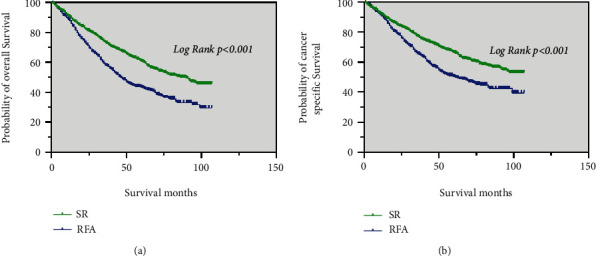
The Kaplan–Meier curve for OS (a) and CSS (b) before PSM for patients with SR and RFA.

Prior to PSM, the median OS of the SR group (86 months) was significantly longer than that of the RFA group (46 months) (*p* < 0.001; Figure 2(a)). The median CSS of patients in the SR was significantly better than that in the RFA group (*p* < 0.001; Figure 2(b)).

**Figure 3:**
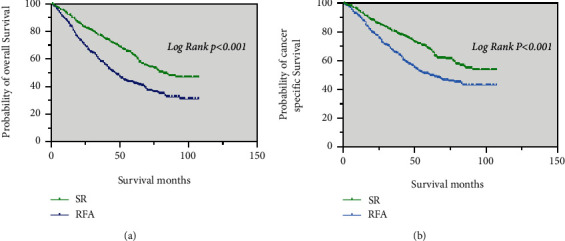
The Kaplan–Meier curve for OS (a) and CSS (b) after PSM for patients with SR and RFA.

After PSM, patients in the SR group (82 months) had a higher median OS than those in the RFA group (46 months) (*p* < 0.001; Figure 3(a)). The SR group had a higher median CSS than those the RFA group (*p* < 0.001; Figure 3(b)).

**Figure 4:**
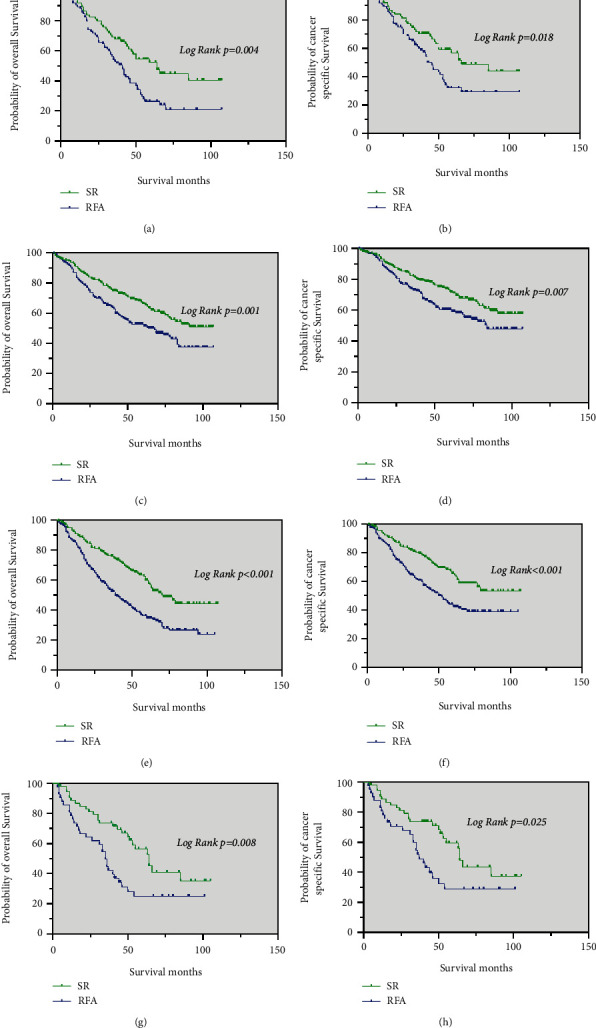
Kaplan–Meier curve of OS and CSS in patients with SR and RFA after PSM—(a and b) patients who received chemotherapy; (c and d) patients with tumor size ≤3 cm; (e and f) patients with tumor size 3–5 cm; (g, h) patients with tumor size >5 cm.

Among patients who received chemotherapy, the median OS was significantly longer in the SR group (64 months) than in the RFA group (41 months) (*p* = 0.004; Figure 4(a)). The median CSS was also significantly longer in the SR group (66 months) than in the RFA group (42 months) (*p* = 0.018; Figure 4(b)).

In those with tumor size <3 cm, the median OS and median CSS of patients in the SR group were significantly better than in the RFA group (*p* = 0.001 and *p* = 0.007, respectively) (Figure 4(c) and 4(d)).

Regarding patients with a single tumor size of 3–5 cm, the median OS was significantly longer in the SR group (71 months) than in the RFA group (39 months) (*p* < 0.001; Figure 4(e)). The median CSS of patients in the SR was significantly better than in the RFA group (*p* < 0.001; Figure 4(f)).

Similar results were noted in patients with tumor sizes >5 cm. The median OS and median CSS were significantly longer in the SR group (64 months, 64 months, respectively) than in the RFA group (35 months, 37 months, respectively) (*p* = 0.008 and *p* = 0.025, respectively) (Figure 4(g) and 4(h)).

**Figure 5:**
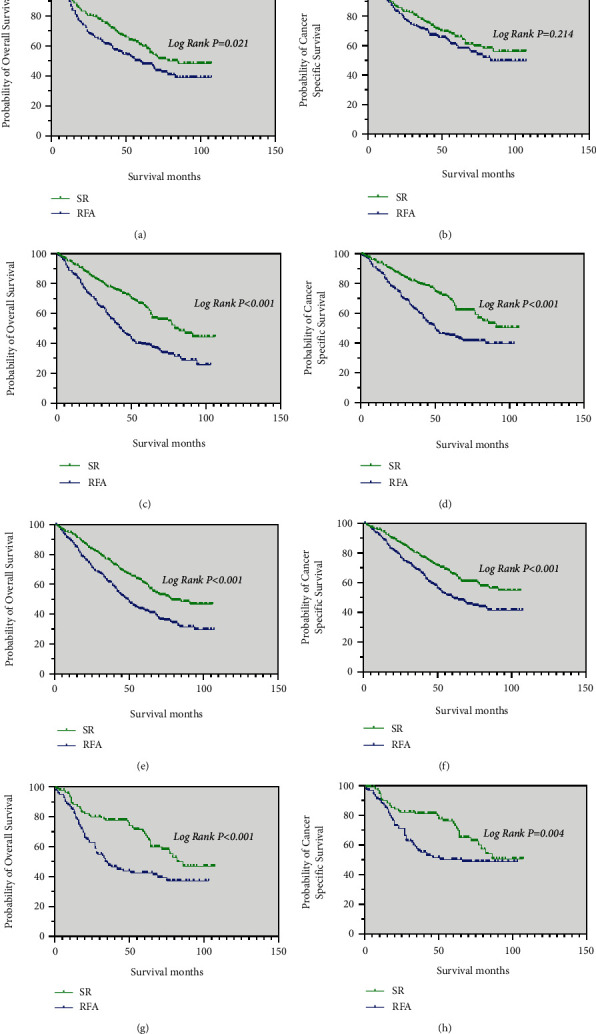
Kaplan–Meier curve of OS and CSS in patients with SR and RFA after PSM—(a and b) patients with an age at diagnosis between 30 and 59 years; (c and d) patients with an age at diagnosis between 60 and 84 years; (e and f) male patients; (g and h) female patients.

In patients with age of diagnosis between 30–59 years, the median OS was significantly longer in the SR group (85 months) than in the RFA group (60 months) (*p* = 0.021). The median CSS was also significantly longer in the SR group than in the RFA group (*p* = 0.214), it is no statistical (Figure 5(a) and 5(b)).

For those with an age at diagnosis between 60 and 84 years, male patients and female patients, the median OS in the SR group (79 months, 79 months, and 82 months, respectively) were longer than that in the RFA group (43 months, 49 months and 34 months, respectively) (*p* < 0.05; Figure 5(c), 5(e), and 5(g)). Meanwhile, the median CSS of these patients in the SR group was significantly better than in the RFA group (*p* < 0.05; Figure 5(d), 5(f), and 5(h)).

**Figure 6:**
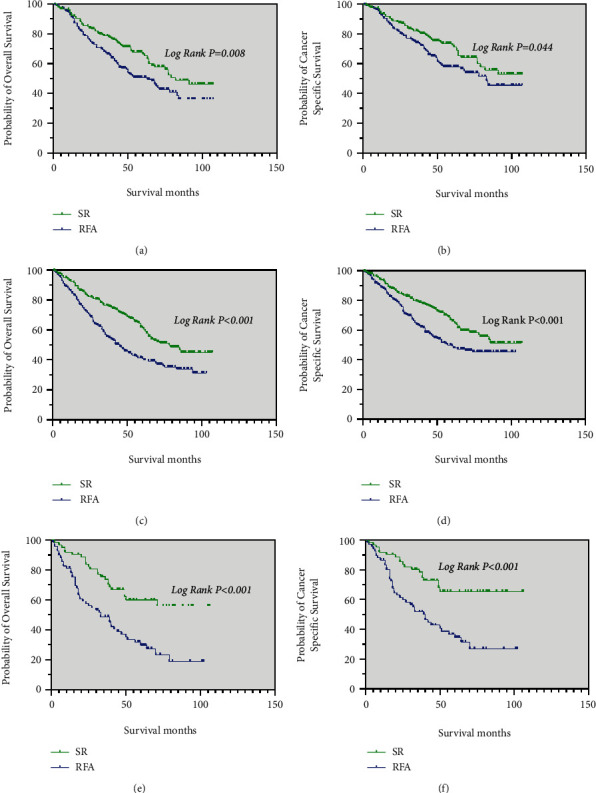
Kaplan–Meier curve of OS and CSS in patients with SR and RFA after PSM—(a and b) patients with Grade I tumors; (c and d) patients with Grade II tumors; (e, f) patients with Grade III and Grade IV tumors.

For patients with grade I tumors and grade II tumors, the median OS in the SR group (82 months, 78 months, respectively) were longer than that in the RFA group (63 months, 45 months, respectively) (*p* < 0.05; Figure 6(a) and 6(c)). The median CSS of these patients in the SR group was significantly better than in the RFA group (*p* < 0.05; Figure 6(b) and 6(d)).

The results of the analysis showed that patients with grade III and grade IV tumors had longer the median OS and median CSS in the SR group than in the RFA group (*p* < 0.001; Figure 6(e) and 6(f)).

### 3.3. Predictors of OS and CSS

Prior to PSM, univariate analysis suggested that compared with RFA, SR was an independent favorable factor for OS (hazard ratio [HR]: 1.628, 95% CI: 1.434–1.847, *p* < 0.001) (Table 2) and CSS (HR: 1.557; 95% CI: 1.351–1.794, *p* < 0.001) (Table 2). Multivariate analysis also showed that compared to RFA, SR was an independent favorable factor for OS (HR: 1.799, 95% CI: 1.523–2.125, *p* < 0.001) (Table 2) and CSS (HR: 1.799; 95% CI: 1.523–2.125, *p* < 0.001) (Table 2). After PSM, similar results were obtained and the univariate analysis showed that compared with RFA, SR was an independent favorable factor for OS (HR: 1.755; 95% CI: 1.470–2.095, *p* < 0.001) (Table 3) and CSS (HR: 1.667; 95% CI: 1.368–2.031, *p* < 0.001) (Table 3). Multivariate analysis showed that compared with RFA, SR was an independent favorable factor for OS (HR: 1.761; 95% CI: 1.474–2.104, *p* < 0.001) (Table 3) and CSS (HR: 1.686; 95% CI: 1.382–2.057, *p* < 0.001) (Table 3).

### 3.4. Prognostic Factors for OS and CSS after PSM

Univariate analysis showed that race, grade, seer stage, chemotherapy, tumor size, AJCC T stage, and treatment was strongly related to OS (Table 3). Multivariate Cox regression analysis showed that the others race (non-black, non-white) (HR: 0.491, 95% CI: 0.382–0.630; *p* < 0.001), grade III (HR: 1.507; 95% CI: 1.137–1.998; *p* = 0.004), grade IV (HR: 3.235; 95% CI: 1.015–10.316; *p* = 0.047), tumor 3–5 cm (HR: 1.340; 95% CI: 1.112–1.614; *p* = 0.002), tumor >5 cm (HR: 1.636; 95% CI: 1.198–2.236; *p* = 0.002), AJCC T stage (HR: 1.606; 95% CI: 1.298–1.986; *p* < 0.001), and treatment modality (HR: 1.761; 95% CI: 1.474–2.104; *p* < 0.001) were significantly associated with OS (Table 3).

In the univariate analysis, age at diagnosis, race, grade, seer stage, chemotherapy, tumor size, AJCC T stage, and treatment were significantly associated with CSS (Table 3). The multivariate Cox regression analysis showed that the others race (HR: 0.519, 95% CI: 0.394–0.683; *p* < 0.001), grade III (HR: 1.566; 95% CI: 1.146–2.142; *p* = 0.005), grade IV (HR: 3.644; 95% CI: 1.130–11.750; *p* = 0.030), tumors of 3–5 cm (HR: 1.329; 95% CI: 1.076–1.641; *p* = 0.008), tumors >5 cm (HR: 1.787; 95% CI: 1.273–2.509; *p* = 0.001), AJCC T stage (HR: 1.718; 95% CI: 1.355–2.178; *p* < 0.001), and treatment (HR: 11.686; 95% CI: 1.382–2.057; *p* < 0.001) were significantly associated with CSS (Table 3).

## 4. Discussion

Our study showed that before and after PSM, SR demonstrated better median OS and CSS than RFA. The results showed that the median OS and median CSS of male and female SR patients were longer than those of RFA patients with tumor sizes <3, 3–5, and >5 cm, age at diagnosis 60–84 years, and grades I–IV tumors. A similar result was also observed in those who received chemotherapy. Several previous studies have recorded similar results, with SR being associated with a longer OS than RFA for patients with small HCC [9, 10]. Mills et al. noted that patients with HCC who undergo SR have a better chance of surviving than those who undergo RFA [11]. Nathan et al. recorded improved survival rates in patients with early HCC who underwent SR [12].

In this study, age at diagnosis, gender, race, grade, seer stage, chemotherapy, tumor size, AJCC T stage, and treatment modality were included in the Cox proportional-hazards model. Before PSM, age at diagnosis, gender, race, grade, chemotherapy, tumor size, AJCC T stage, and treatment modality influenced OS and CSS. After PSM, OS and CSS were only associated with race, grade, tumor size, AJCC T stage, and treatment modality. Thus, we concluded that patients who had single HCCs that were poorly differentiated, with higher AJCC T stages and greater sizes, had worse prognoses, and this is in agreement with other population-based studies [13]. Margonis et al. indicated that in lesions with poorly differentiated HCC, the prognosis might be less favorable [14]. A prospective study by Camma et al. showed that patients with HCC whose tumors were <3 cm had better outcomes after treatment [15]. Likewise, our study showed that tumor size <3 cm was associated with superior survival compared to tumor size of 3–5 cm. The prognosis of most solid tumors is predicted based on the AJCC TNM system [16], as our results reflect. Citterio et al. noted that in patients with single HCC and no lymph nodes or distant metastases, SR is frequently performed [17]. SR is recommended as the first-line treatment for patients with very early-stage HCC since it has a better OS than RFA [9]. The results of our study are similar to some previous studies [18, 19] that showed that SR provides better therapeutic effect than RFA for small single HCC. The present study has some limitations. First, due to its retrospective design, selection bias was inevitable. Thus, we attempted to reduce the selection bias by PSM, which ensured a better balance in the baseline characteristics between the two groups. Second, the hepatic function and health of the patients were unknown because the SEER database did not contain this information. This lack of information might have influenced the results. However, previous studies have shown that those with a single HCC who undergo SR had better survival outcomes compared with those who received RFA [20]. The results of our study are similar to those of previous studies, but the SEER database did not contain HCC predisposing factors, such as hepatitis or steatohepatitis. There was only macroscopic information regarding the treatment methods in the SEER database, which did not include chemotherapy regimens and surgery programs. Considering this, it is necessary to conduct a prospective randomized trial to confirm these results in future work.

## 5. Conclusion

In this population-based study, patients with single HCC who underwent SR had better OS and CSS compared to those who received RFA, indicating that SR should be used as a first-line treatment in such cases. The research showed that the prognosis was poorer for patients who had single HCC with poor differentiation, higher AJCC T stages, and greater tumor sizes.

## Figures and Tables

**Table 1:** Characteristics of patients.

	Before PSM (*N* = 2175)				After PSM (*N* = 1048)			
Characteristics	SR (*N* = 1498)	RFA (*N* = 677)	*P*	SMD	SR (*N* = 524)	RFA (*N* = 524)	*P*	SMD
Age of diagnosis (%)			0.528	0.030			0.653	0.032
30–59 years	530 (35.4)	230 (34.0)			193 (36.8)	185 (35.3)		
60–84 years	968 (64.6)	447 (66.0)			339 (64.7)	331 (63.2)		
Gender (%)			0.434	0.039			0.615	0.035
Male	1085 (72.4)	502 (74.2)			392 (74.8)	400 (76.3)		
Female	413 (27.6)	175 (27.6)			132 (25.2)	124 (23.7)		
Race (%)			<0.001	0.322			0.674	0.002
White	808 (54.0)	463 (68.4)			345 (65.8)	349 (66.6)		
Black	207 (13.8)	75 (11.1)			66 (12.6)	57 (10.9)		
Others	483 (32.2)	139 (20.5)			113 (21.6)	118 (22.5)		
Grade (%)			<0.001	0.474			0.851	0.017
I	326 (21.8)	267 (39.4)			179 (34.2)	179 (34.2)		
II	821 (54.8)	334 (49.3)			283 (54.0)	276 (52.7)		
III	324 (21.6)	74 (11.0)			59 (11.3)	67 (12.8)		
IV	27 (1.8)	2 (0.3)			3 (0.5)	2 (0.3)		
SEER stage (%)			<0.001	0.147			1	<0.001
Localized	1381 (92.2)	591 (87.3)			481 (91.8)	481 (91.8)		
Regional	117 (7.8)	86 (12.7)			43 (8.2)	43 (8.2)		
Chemotherapy (%)			<0.001	0.340			0.440	0.044
No	1343 (89.7)	507 (74.9)			449 (85.7)	439 (83.8)		
Yes	155 (10.3)	170 (25.1)			75 (14.3)	85 (16.2)		
Tumor size (%)			<0.001	0.951			0.067	0.034
<3 cm	388 (25.9)	368 (54.4)			277 (52.9)	254 (48.5)		
3–5 cm	566 (37.8)	266 (39.3)			193 (36.8)	228 (43.5)		
>5 cm	544 (36.3)	43 (6.3)			54 (10.3)	42 (8.0)		
AJCC T (%)			0.643	0.023			1	0.004
T1	1082 (72.2)	482 (71.2)			407 (77.7)	408 (77.9)		
T2	416 (27.8)	195 (28.8)			117 (22.3)	116 (22.1)		

**Table 2:** Predictors for overall survival and cancer-specific survival prior to PSM.

	Overall survival				Cancer-specific survival			
	Univariate analysis		Multivariate Analysis		Univariate analysis		Multivariate analysis	
Characteristics	HR (95% CI)	*P*	HR (95% CI)	*P*	HR (95% CI)	*P*	HR (95% CI)	*P*
Age of diagnosis								
30–59 years	Reference		Reference		Reference		Reference	
60–84 years	1.228 (1.075, 1.402)	0.002	1.186 (1.038, 1.357)	0.012	1.235 (1.065, 1.433)	0.005	1.189 (1.024, 1.381)	0.023
Gender								
Male	Reference		Reference		Reference		Reference	
Female	0.816 (0.705, 0.943)	0.006	0.832 (0.719, 0.964)	0.014	0.815 (0.693, 0.958)	0.013	0.826 (0.702, 0.972)	0.022
Race								
White	Reference		Reference		Reference		Reference	
Black	1.048 (0.877,1.251)	0.608	1.093 (0.914,1.306)	0.329	1.052 (0.862,1.283)	0.62	1.100 (0.901,1.342)	0.35
Others	0.576 (0.492, 0.674)	<0.001	0.601 (0.513, 0.704)	<0.001	0.611 (0.514, 0.725)	<0.001	0.633 (0.532, 0.754)	<0.001
Grade								
I	Reference		Reference		Reference		Reference	
II	1.098 (0.946, 1.275)	0.22	1.159 (0.996, 1.349)	0.057	1.122 (0.948, 1.328)	0.18	11.168 (0.985, 1.386)	0.075
III	1.371 (1.142, 1.647)	0.001	1.592 (1.318, 1.924)	<0.001	1.500 (1.225, 1.836)	<0.001	1.705 (1.383, 2.102)	<0.001
IV	1.329 (0.790, 2.237)	0.284	1.523 (0.897, 2.586)	0.12	1.478 (0.843, 2.590)	0.172	1.571 (0.888, 2.782)	0.121
SEER stage								
Localized	Reference		Reference		Reference		Reference	
Regional	1.417 (1.164, 1.725)	0.001	1.109 (0.900, 1.366)	0.331	1.569 (1.272, 1.937)	<0.001	1.204 (0.963, 1.505)	0.103
Chemotherapy								
No	Reference		Reference		Reference		Reference	
Yes	1.538 (1.315, 1.797)	<0.001	1.186 (1.007, 1.397)	0.041	1.667 (1.407, 1.975)	<0.001	1.273 (1.065, 1.521)	0.008
Tumor size								
<3 cm	Reference		Reference		Reference		Reference	
3–5 cm	1.194 (1.030, 1.383)	0.018	1.319 (1.134, 1.535)	<0.001	1.218 (1.031, 1.439)	0.02	1.335 (1.125, 1.583)	0.001
>5 cm	1.259 (1.074, 1.477)	0.005	1.616 (1.352, 1.931)	<0.001	1.401 (1.175, 1.671)	<0.001	1.783 (1.463, 2.171)	<0.001
AJCC T								
T1	Reference		Reference		Reference		Reference	
T2	1.527 (1.340, 1.740)	<0.001	1.436 (1.251, 1.648)	<0.001	1.657 (1.435, 1.912)	<0.001	1.528 (1.313, 1.777)	<0.001
Treatment								
Surgical resection	Reference		Reference		Reference		Reference	
Radiofrequency ablation	1.628 (1.434, 1.847)	<0.001	1.828 (1.577, 2.120)	<0.001	1.557 (1.351, 1.794)	<0.001	1.799 (1.523, 2.125)	<0.001

**Table 3:** Predictors for overall survival and cancer-specific survival after PSM.

	Overall survival				Cancer-specific survival			
	Univariate analysis		Multivariate analysis		Univariate analysis		Multivariate analysis	
Characteristics	HR (95% CI)	*P*	HR (95% CI)	*P*	HR (95% CI)	*P*	HR (95% CI)	*P*
Age of diagnosis								
30–59 years	Reference				Reference		Reference	
60–84 years	1.129 (0.941,1.354)	0.193			1.201 (0.977,1.476)	0.082	1.198 (0.971,1.477)	0.092
Gender								
Male	Reference				Reference			
Female	0.990 (0.807,1.215)	0.925			1.001 (0.796, 1.259)	0.993		
Race								
White	Reference		Reference		Reference		Reference	
Black	1.008 (0.777, 1.308)	0.952	1.005 (0.773, 1.306)	0.97	0.997 (0.742, 1.339)	0.982	0.984 (0.731, 1.324)	0.914
Others	0.506 (0.395, 0.648)	<0.001	0.491 (0.382, 0.630)	<0.001	0.541 (0.412, 0.710)	<0.001	00.519 (0.394, 0.683)	<0.001
Grade								
I	Reference	Reference	Reference	Reference				
II	1.171 (0.964, 1.423)	0.111	1.144 (0.941, 1.391)	0.178	1.185 (0.952, 1.476)	0.129	1.150 (0.922, 1.434)	0.214
III	1.407 (1.062, 1.863)	0.017	1.507 (1.137, 1.998)	0.004	1.491 (1.092, 2.035)	0.012	1.566 (1.146, 2.142)	0.005
IV	2.136 (0.681, 6.696)	0.193	3.235 (1.015, 10.316)	0.047	2.788 (0.887, 8.769)	0.079	3.644 (1.130, 11.750)	0.03
SEER stage								
Localized	Reference		Reference		Reference		Reference	
Regional	1.329 (0.993, 1.780)	0.056	0.942 (0.687, 1.290)	0.707	1.380 (0.999, 1.906)	0.051	0.932 (0.657, 1.322)	0.692
Chemotherapy								
No	Reference		Reference		Reference		Reference	
Yes	1.433 (1.149,1.786)	0.001	1.148 (0.908,1.451)	0.249	1.568 (1.233,1.994)	<0.001	1.242 (0.959,1.608)	0.1
Tumor size								
<3 cm	Reference		Reference		Reference		Reference	
3–5 cm	1.427 (1.187, 1.715)	<0.001	1.340 (1.112, 1.614)	0.002	1.450 (1.178, 1.785)	<0.001	1.329 (1.076, 1.641)	0.008
>5 cm	1.540 (1.149, 2.063)	0.004	1.636 (1.198, 2.236)	0.002	1.772 (1.294, 2.426)	<0.001	1.787 (1.273,2.509)	0.001
AJCC T								
T1	Reference		Reference		Reference		Reference	
T2	1.641 (1.354, 1.989)	<0.001	1.606 (1.298, 1.986)	<0.001	1.722 (1.390, 2.133)	<0.001	1.718 (1.355, 2.178)	<0.001
Treatment								
Surgical resection	Reference		Reference		Reference		Reference	
Radiofrequency ablation	1.755 (1.470, 2.095)	<0.001	1.761 (1.474, 2.104)	<0.001	1.667 (1.368, 2.031)	<0.001	1.686 (1.382, 2.057)	<0.001

## Data Availability

All data were acquired from the SEER database.
